# Predominant but Previously-overlooked Prokaryotic Drivers of Reductive Nitrogen Transformation in Paddy Soils, Revealed by Metatranscriptomics

**DOI:** 10.1264/jsme2.ME16179

**Published:** 2017-04-22

**Authors:** Yoko Masuda, Hideomi Itoh, Yutaka Shiratori, Kazuo Isobe, Shigeto Otsuka, Keishi Senoo

**Affiliations:** 1 Department of Applied Biological Chemistry, Graduate School of Agricultural and Life Sciences, The University of Tokyo1–1–1 Yayoi, Bunkyo-ku, Tokyo 113–8657Japan; 2 Bioproduction Research Institute, National Institute of Advanced Industrial Science and Technology (AIST), Hokkaido Center2–17–2–1 Tsukisamu-higashi, Toyohira, Sapporo 062–8517Japan; 3 Niigata Agricultural Research Institute857 Nagakuramachi, Nagaoka, Niigata 940–0826Japan

**Keywords:** paddy soils, metatranscriptomics, denitrification, dissimilatory nitrate reduction to ammonium, nitrogen fixation

## Abstract

Waterlogged paddy soils possess anoxic zones in which microbes actively induce reductive nitrogen transformation (RNT). In the present study, a shotgun RNA sequencing analysis (metatranscriptomics) of paddy soil samples revealed that most RNT gene transcripts in paddy soils were derived from *Deltaproteobacteria*, particularly the genera *Anaeromyxobacter* and *Geobacter*. Despite the frequent detection of the rRNA of these microbes in paddy soils, their RNT-associated genes have rarely been identified in previous PCR-based studies. This metatranscriptomic analysis provides novel insights into the diversity of RNT microbes present in paddy soils and the ecological function of *Deltaproteobacteria* predominating in these soils.

Paddy soils are characterized by temporal anaerobic conditions caused by waterlogging, and the active occurrence of anaerobic biogeochemical processes (9). Among these active processes, biological reductive nitrogen transformation (RNT), *i.e.*, denitrification (NO_3_^−^→NO_2_^−^→NO→N_2_O→N_2_), dissimilatory nitrate reduction to ammonium (DNRA; NO_3_^−^→NO_2_^−^→NH_4_^+^), and nitrogen fixation (N_2_→NH_4_^+^) contribute to less leaching of nitrogen pollutants (NO_3_^−^, NO_2_^−^, and N_2_O) into the environment and the greater retention of nitrogen-based nutrients (NH_4_^+^) for rice plants in waterlogged paddy soils than in upland soils (8, 22). Therefore, the identification of microbial drivers of RNT in paddy soils is important for successful rice production with minimal environmental nitrogen burden.

However, a comprehensive understanding of the RNT microbial community has not yet been achieved. In order to investigate RNT microbes in paddy soils, genes encoding the enzymes that catalyze each reaction have been assessed via PCR-based culture-independent methods, as represented by a clone library analysis (13, 24). Recent studies based on bacterial genomics reported that the diversity of microbes harboring RNT genes is greater than previously considered; PCR-based methods have underrepresented this diversity because of mismatches in the sequences of the primers used (5, 10, 21), indicating the need for alternative methods without a PCR bias. Furthermore, simultaneous assessments of microbes involved in denitrification, DNRA, and nitrogen fixation in a single paddy field have not yet been performed. Moreover, limited information is available on the transcriptional profiles *in situ* of RNT microbes in paddy soils because of the small number of field studies conducted based on soil RNA, which directly implicates RNT microbial activity. In the present study, we investigated RNT-associated microbial diversity in paddy soils via a shotgun RNA sequencing analysis without any prior PCR preparation (metatranscriptomics).

In order to obtain a more complete understanding of paddy soils with various biogeochemical properties spatially and seasonally (9, 12), soil RNA extracted from paddy soils in shallow (S1, S3) and deep (S2, S4) layers under waterlogged (S1, S2) and drained (S3, S4) conditions (Fig. S1) were subjected to a metatranscriptomic analysis using an Illumina MiSeq sequencer (Illumina, San Diego, CA, USA). The sequences of RNT genes were retrieved from the metatranscriptomic libraries obtained and taxonomically annotated through a tandem similarity search with the blat and blast programs (full methods in Supplementary information).

Four reactions crucial to denitrification are catalyzed by the following enzymes: NO_3_^−^ reductase (Nar), NO_2_^−^ reductase (Nir), NO reductase (Nor), and N_2_O reductase (Nos). The *nar* transcripts detected in all soil samples using metatranscriptomics were related to those of *Deltaproteobacteria*, *Betaproteobacteria*, *Alphaproteobacteria*, *Gammaproteobacteria*, and *Acidobacteria* (Fig. 1A), suggesting that these bacterial groups are involved in the reduction of NO_3_^−^ to NO_2_^−^. The *nir* transcripts were mostly derived from *Betaproteobacteria*, *Gammaproteobacteria*, and *Alphaproteobacteria* (Fig. 1A), the members of which include common denitrifiers (5); these were also frequently detected in the same paddy soils in our previous PCR-based survey (24). Furthermore, *nor* and *nos* transcripts were predominantly detected in *Deltaproteobacteria* (Fig. 1A), the transcripts of which were rarely detected via previous PCR assays (2, 24). Successive denitrification steps were considered to be associated with common denitrifiers, such as *Betaproteobacteria*, *Gammaproteobacteria*, *Alphaproteobacteria*, and *Actinobacteria*, which harbor *nir*, *nor*, and/or *nos* (18). However, the metatranscriptomic data obtained in the present study suggested that the reduction of NO_2_^−^ into NO was driven by these denitrifiers, and that the reduction of NO and N_2_O was mainly progressed by non-denitrifiers such as *Deltaproteobacteria*, *Bacteroidetes*, *Acidobacteria*, and *Verrucomicrobia*, which harbor *nor* and/or *nos*, but not *nir* (5). Thus, paddy soil denitrification appears to be a cooperative process by each nitrogen oxide reducer, *i.e.*, NO_2_^−^ reducers (denitrifiers) and NO/N_2_O reducers (non-denitrifiers), similar to nitrification (NH_4_^+^→NO_2_^−^→NO_3_^−^) orchestrated by NH_4_^+^-oxidizing bacteria/archaea and NO_2_^−^-oxidizing bacteria (6). These inferences in the denitrification process may be verified using co-culture experiments on denitrifiers and non-denitrifiers.

DNRA, another NO_3_^−^ reduction process, is catalyzed by Nar and NH_4_^+^-forming NO_2_^−^ reductase (Nrf). Most of the *nrf* transcripts belong to *Deltaproteobacteria*, while some belong to *Verrucomicrobia* (Fig. 1A). Together with the frequent detection of *nar* transcripts derived from *Deltaproteobacteria* as described above, *Deltaproteobacteria* appear to mainly contribute to DNRA dynamics in paddy soils. Although DNRA has been geochemically detected in paddy soils (1, 23), limited information is available on DNRA microbial diversity. To the best of our knowledge, the present study is the first to attempt to identify the key player groups in DNRA in paddy soils.

Diazotrophs harboring nitrogenase (Nif) drive nitrogen fixation. The taxonomic composition of *nif* transcripts was dominated by *Deltaproteobacteria* (Fig. 1A), indicating that *Deltaproteobacteria* represents a key player group in nitrogen fixation. Rhizospheric *Alphaproteobacteria*, *Betaproteobacteria*, and *Gammaproteobacteria* and phototrophic *Cyanobacteria* were considered to be key diazotrophs in paddy soils (13, 18). However, we detected more *nif* transcripts in *Deltaproteobacteria* than in these well-known diazotrophs; our results were consistent with a recent metatranscriptomic analysis based on a microcosm study on Italian paddy soils (11).

The abundance of RNT genes derived from *Deltaproteobacteria*, as described above, was also demonstrated in a shotgun DNA sequencing analysis (metagenomics) (Fig. S2A). Additionally, the microbial community structure based on rRNA gene/transcript sequences showed that *Deltaproteobacteria* is a major group in paddy soil microbes (Fig. 1A, S2A). These results support *Deltaproteobacteria* being a key player group driving RNT in paddy soils.

Further analyses on *Deltaproteobacteria* at the genus level revealed the consistent detection of RNT gene transcripts in metatranscriptomic data derived from the genera *Anaeromyxobacter* and *Geobacter* (Fig. 1B), as well as their RNT genes in metagenomic data (Fig. S2B). These genera represent obligate anaerobes and metal reducers predominating in paddy soils (7, 20) and exhibit some RNT activities *in vitro* (summarized in Table S1). Although the nitrogen fixation activity of *Anaeromyxobacter* has yet to be characterized, the genomes of some *Anaeromyxobacter* spp. conserve a similar *nif* cluster to that of *Geobacter* spp. exhibiting nitrogen fixation activity (Fig. S3). Together with the detection of the *nif* transcripts of *Anaeromyxobacter* in this study, it is plausible that *Anaeromyxobacter* spp. perform nitrogen fixation. However, in contrast to *Anaeromyxobacter* and *Geobacter* rRNA genes, their RNT genes have rarely been detected in paddy soil samples using PCR-based techniques (4, 24). Thus, the putative role of these genera in the RNT process has received little attention despite their predominance in paddy soils. The limited coverage of RNT gene-specific PCR primers used in previous studies may have led to the oversight of these genera (10, 17); additionally, the GC content may be implicated because the *nor*/*nos*/*nrf*/*nif* of *Anaeromyxobacter* spp. showed markedly higher GC contents than the rRNA genes and *nor*/*nos*/*nrf*/*nif* of other bacteria (Table S2). Even improved *nos* universal primers, which have enabled lower rates of sequence mismatches, were unable to amplify *Anaeromyxobacter nos* (10). Therefore, a metatranscriptomic analysis represents a more effective approach to examine the diversity of functional microbes, without any PCR bias arising from the high GC content of target genes as well as primer limitations.

*Anaeromyxobacter* and *Geobacter*, which have frequently been detected in Japanese, Chinese, and Italian paddy soils (4, 11, rRNA data in Fig. 1B), predominate more in paddy soils than in upland soils, as confirmed by the present study (Fig. 2A, B; Table S3). Their universal distribution and predominance in paddy soils support *Anaeromyxobacter* and *Geobacter* being the key RNT players in paddy soils. Furthermore, the predominance of these genera was found in river sediments (Fig. 2A, B; Table S3); the RNT genes of *Anaeromyxobacter* were frequently and globally detected in upland soil environments in recent shotgun metagenomics studies (14, 15), indicating the contribution of these bacteria to RNT not only in paddy soils, but also in other environments. The further application of metatranscriptomics across different environments will expand our knowledge on the diversity of RNT microbes in nature as well as the ecological function of *Deltaproteobacteria* in soil environments.

Previous studies on paddy soils identified the predominance of *Deltaproteobacteria* and their ecological roles in dissimilatory metal reduction, sulfur/sulfate reduction, and hydrogen production (7, 9, 19). Although genomic studies showed the ubiquitous possession of the RNT genes of *Deltaproteobacteria*, the association of RNT with *Deltaproteobacteria* has not been considered because of the rare detection of their RNT genes in soil environments through PCR-based analyses. The present study revealed the novel ecological functions of *Anaeromyxobacter* and *Geobacter* within *Deltaproteobacteria* dominating in paddy soils, namely, RNT, denitrification support, and NH_4_^+^ production via DNRA and nitrogen fixation (Fig. 3).

## Supplementary information



## Figures and Tables

**Fig. 1:**
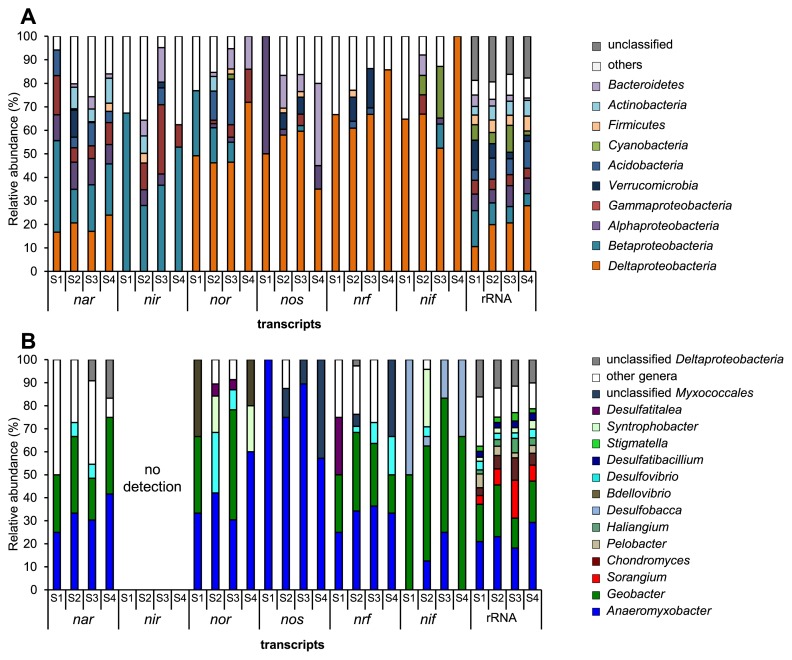
Microbial diversity of RNT gene transcripts and rRNA. Taxonomic distribution of *nar*, *nir*, *nor*, *nos*, *nrf*, and *nif* transcripts, and rRNA at the phylum and proteobacterial class level (A), and deltaproteobacterial genus level (B). Sample IDs indicate data derived from paddy soils in shallow (S1, S3) and deep (S2, S4) layers under waterlogged (S1, S2) and drained (S3, S4) conditions. Data represent the mean of triplicates.

**Fig. 2:**
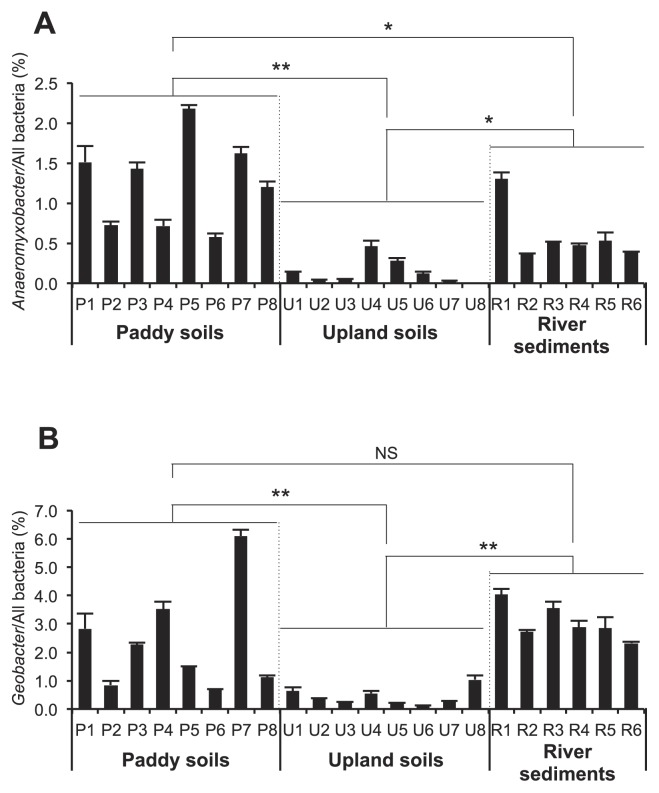
Distribution of *Anaeromyxobacter* and *Geobacter* in various soil environments. Proportions of *Anaeromyxobacter* (A) and *Geobacter* (B) against all bacteria estimated by a quantitative PCR method. The mean±SD is shown (*n*=3). The paddy soil sample P3 was collected from the same paddy field used for the metatranscriptomic analysis in this study. Asterisks indicate significant differences (Mann-Whitney *U* test; *, *p*<0.01; **, *p*<0.001); NS, not significant. Details of soil samples and qPCR data are summarized in Table S3.

**Fig. 3:**
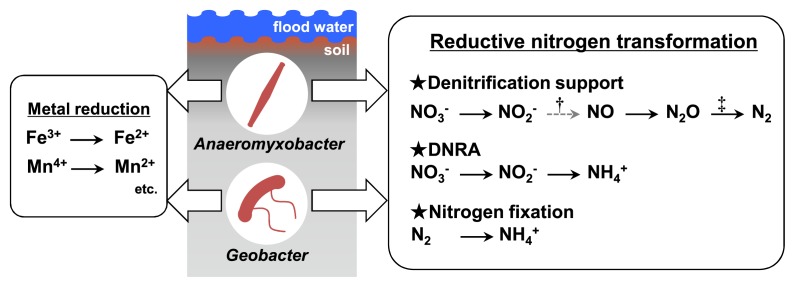
Ecological functions of *Anaeromyxobacter* and *Geobacter*, belonging to *Deltaproteobacteria*, predominant in paddy soils, expanded by metatranscriptomics in this study. *Anaeromyxobacter* and *Geobacter*, ubiquitously predominant in paddy soils, are key player groups in the reduction of iron and manganese, which actively progresses in paddy soils soon after waterlogging (3, 4, 7, 9, 11, 20, Fig. 1B, S2B). Metatranscriptomics in this study suggested that these genera also associate with reductive nitrogen transformation, *i.e.*, denitrification, DNRA, and nitrogen fixation. Sketches of *Anaeromyxobacter* and *Geobacter* are based on electron microscope photographs published elsewhere (3, 16). †, NO_2_^−^ reduction to NO is driven by common denitrifiers within *Alphaproteobacteria*, *Betaproteobacteria*, and *Gammaproteobacteria* (5), not by *Anaeromyxobacter* and *Geobacter* within *Deltaproteobacteria*; ‡, *Anaeromyxobacter* reduce N_2_O to N_2_, whereas *Geobacter* do not (Table S1).
