# Supplementary material for: Predominant but Previously-overlooked Prokaryotic Drivers of Reductive Nitrogen Transformation in Paddy Soils, Revealed by Metatranscriptomics
Source: Microbes Environ. 2017 Apr 22;32(2):180–3. doi: 10.1264/jsme2.ME16179 (PMC5478542; doi:10.1264/jsme2.ME16179)
Supplement: Supplementary file 1 [file 32_180_s1.pdf]

Supplementary information

Materials and methods

Study field and soil sampling

The paddy field examined in this study is located at Niigata Agricultural Research Institute (Nagaoka, Niigata, Japan: 37°26'N, 138°52'E). The soil type, cropping history, and soil physicochemical properties of the field were described previously (Itoh *et al.*, 2013). Field managements such as tilling, water management, fertilization, herbicide spraying in 2012, when we examined in this study, was basically same as those reported in our previous study (Itoh *et al.*, 2013).

Six transparent acrylic cores (10 × 15 cm; inner diameter × depth) were randomly placed in the plow layer (0 to 10 cm) at the furrow of the paddy field just after puddling on May 7th in 2012. Soil samples were collected at 2 time points, Jun 18th (waterlogged condition), and Oct 1st (drained condition), which showed the lowest and highest Eh in cultivation season, respectively (Fig. S1). At each sampling event, 3 cores were collected after removing the surface water, if any, and immediately frozen in liquid nitrogen, transported to the laboratory with dry ice, and stored at -80°C until use. Shallow and deep layers from a depth of 0 to 1 cm and 5 to 7 cm, respectively, were cut out from each frozen cylindrical soil by a sterilized knife. Each of soil samples contained three independent replicates.

Soil and sediment samples from a depth of 0 to 10 cm used for quantitative

PCR (qPCR) analyses were collected from paddy fields, upland fields, and river in various districts across the Japan (Table S3), and stored at -80°C until DNA extraction.

Preparation of soil RNA and DNA

Four soil samples with triplicates (12 samples in total), which were shallow (S1, S3) and deep (S2, S4) layer samples obtained from soil cores collected in waterlogged (S1, S2) and drained (S3, S4) seasons, were subjected to RNA and DNA extraction and applied for metatranscriptomics and metagenomics in this study. Soil RNA and DNA were simultaneously extracted from 2 g (wet weight) of each soil sample using PowerSoil Total RNA Isolation Kit (MoBio Laboratories, Solana Beach, CA, USA) with PowerSoil DNA Elution Accessory Kit (MoBio Laboratories) (Itoh *et al.*, 2013). Crude RNA was purified using the TURBO DNA-free Kit (Applied Biosystems, Foster City, CA, USA) and RNA Clean & Concentrator (Zymo Research) as described previously (Itoh *et al.*, 2013). No contamination of DNA in prepared RNA was confirmed by PCR amplification using specific primers for bacterial 16S rRNA gene as described in *qPCR* section below. Crude DNA was purified using the RNase A (TAKARA BIO INC., Otsu, Shiga, Japan) and DNA Clean & Concentrator according to the manufacture's instruction (Zymo Research). Purity of the prepared RNA and DNA were determined using the NanoDrop ND-1000 spectrophotometer (NanoDrop Technologies, Wilmington, DE, USA) as described previously (Itoh *et al.*, 2013), and

RNA integrity was confirmed using the Agilent 2100 Bioanalyzer (Agilent Technologies, Palo Alto, CA, USA) with the RNA 6000 Pico Kit (Agilent Technologies). The quantity of the prepared RNA and DNA were measured using Qubit 2.0 Fluorometer (Invitrogen, Carlsbad, CA, USA) with Qubit RNA Assay Kits (Invitrogen) and Qubit dsDNA HS Assay Kits (Invitrogen), respectively.

For qPCR analyses, soil and sediments DNA were extracted from 0.5 g (wet weight) of each sample using ISOIL for Beads Beating (Nippon Gene, Toyama, Japan) added with 0.02 g skim milk for improvement of extraction efficiency (Takada-Hoshino *et al.*, 2004). Purity and quantity of the prepared DNA was determined using the NanoDrop ND-1000 spectrophotometer (NanoDrop Technologies).

Shotgun metatranscriptomic and metagenomic sequencing

In order to perform the simultaneous assessment of community structure and function of soil microbes based on rRNA and mRNA (Urich et al., 2008), total RNA was subjected to construction of complementary DNA (cDNA) libraries using the NEBNext Ultra RNA Library Prep Kit for Illumina (New England Biolabs Inc., Beverly, Massachusetts, USA). For metagenomic sequencing, prepared soil DNA was sheared into 300 bp fragments by the Covaris M220 (Covaris Inc., Woburn, MA, USA), and then DNA libraries for metagenomic sequencing was constructed using the fragmented DNA with the NEBNext Ultra DNA Library Prep Kit for Illumina (New England

Biolabs Inc.). Size distribution of cDNA and DNA libraries were checked on the Agilent 2100 Bioanalyzer (Agilent Technologies). Paired-end sequencing for all libraries was performed on an Illumina MiSeq sequencer (Illumina, San Diego, CA, USA) using a MiSeq Reagent kit v2 (Illumina) according to the manufacture's instruction.

Informatics and statistics

Resulting paired-end sequences were joined together as described previously (Itoh *et al.*, 2014). Fastq-formatted data for the combined sequences were converted to fasta-formatted data with a Q-score cutoff of >30 using a PRINSEQ lite ver. 0.20.3 (Schmieder and Edwards, 2011). Ribosomal RNA sequences in metatranscriptomic data were determined by BLAT search against the M5RNA database (e-value of $<10^{-5}$, alignment length of >50 bp) on the MG-RAST server ver. 3.6 (Meyer *et al.*, 2008). Protein-coding genes transcripts were retrieved from metatrascriptomic data through BLAT search against the M5nr database (Wilke *et al.*, 2012) (e-value of $<10^{-5}$, alignment length of >30 aa) on the MG-RAST server ver. 3.6 (Meyer *et al.*, 2008). From all of these assigned transcripts, sequences assigned as functional genes involved in reductive nitrogen transformation, i.e. *nar*, *nir*, *nor*, *nos*, *nrf*, and *nif* were collected and compared to the NCBI nr database in December 2015 by BLASTX search for more precise assignment (e-value of $<10^{-5}$). Domain structure of query-tophit references was

confirmed by NCBI conserved domain search (Marchler-Bauer *et al.*, 2015), in order to assign based on not only reference name but also domain structure. To minimize the bias owing to differences of sequencing depth among libraries, the trimmed mean of M values (TMM) normalization method available in the edge R Bioconductor package was applied and used to calculate the normalized ratio of taxonomic composition of rRNA and functional genes involved in reductive nitrogen transformations (Robinson *et al.*, 2010). Mann-Whitney *U* test was performed using R software ver. 3.0.1 (R Development Core Team, 2008) to analyze qPCR results.

qPCR

qPCR was performed to amplify 16S rRNA genes of all bacteria, *Geobacter*, and *Aaneromyxobacter* using the StepOnePlus System (Life Technologies, Carlsbad, CA, USA) with the SYBR Premix EX Taq II (TAKARA BIO INC.). The reaction mixture was comprised of 1× SYBR Premix Ex Taq II, 0.2 μM forward and reverse primer pairs (338F and 518R for all bacteria (Klammer, *et al.*, 2008), 494F and Geo825R for *Geobacter* (Holmes *et al.*, 2002; Anderson *et al.*, 1998), Ade399F and Ade466R for *Anaeromyxobacter* (Thomas, 2009)), 0.5 μg/μl BSA, and soil DNA as a template. The PCR conditions were as follows: initial denaturation at 95°C for 30 s, followed by 40 cycles of 95°C for 5 s, 55°C for 30 s (for all bacteria) or 50°C for 20 s (for *Geobacter* and *Anaeromyxobacter*), and 72°C for 30 s. Copies numbers of 16S

rRNA genes of all bacteria, *Geobacter*, and *Anaeromyxobacter* were calculated on the basis of the standard curve constructed using a dilution series of the targeted PCR products of *Pseudomonas stutzeri* JCM 5965, *Geobacter sulfurreducens* JCM 18752, or *Anaeromyxobacter dehalogenas* 2CP-C ATCC BAA-259, respectively.

Nucleotide sequence accession number

All of metatranscriptomic and metagenomic sequences reported in this study were deposited in the MG-RAST database (http://metagenomics.anl.gov/, Meyer *et al.*, 2008). Deposit IDs were summarized in Tables S4 and S5.

References

Anderson, R.T., J. N. Rooney-Verga, C.V. Gaw, and D.R. Lovley. 1998. Anaerobic benzene oxidation in the Fe(III) reduction zone of petroleum-contaminated aquifers. Environ. Sci. Technol. 32: 1222–1229.

Bazylinski, D.A., A.J. Dean, D. Schüler, E.J. Phillips, and D.R. Lovley. 2000. N_2-dependent growth and nitrogenase activity in the metal-metabolizing bacteria, *Geobacter* and *Magnetospirillum* species. Environ. Microbiol. 2: 266–273.

Klammer, S., B. Knapp, H. Insam, M.T. Dell'Abate, and M. Ros. 2008. Bacterial community patterns and thermal analyses of composts of various origins. Waste. Manag. Res. 26: 173–187.

Itoh, H., S. Ishii, Y. Shiratori, K. Oshima, S. Otsuka, M. Hattori, and K. Senoo. 2013. Seasonal transition of active bacterial and archaeal communities in relation to water management in paddy soils. Micobes Environ. 28: 370–380.

Itoh, H., M. Aita, A. Nagayama, X.Y. Meng, Y. Kamagata, R. Navarro, T. Hori, S. Ohgiya, and Y. Kikuchi. 2014. Evidence of environmental and vertical transmission of *Burkholderia Symbionts* in the oriental chinch bug, *Cavelerius saccharivorus* (Heteroptera: Blissidae). Appl. Environ. Microbiol. 80: 5974–5083.

Holmes, D.E., K.T. Finneran, R.A. O'neil, and D.R.Lovley. 2002. Enrichment of members of the family *Geobacteraceae* associated with stimulation of dissimilatory metal reduction in uranium-contaminated aquifer sediments. Appl. Environ.

Microbiol. 68: 2300–2306.

Marchler-Bauer, A., M.K. Derbyshire, N.R. Gonzales, et al. 2015. CDD: NCBI's conserved domain database. Nucleic Acids Res. 43: D222–226.

Methé, B. A., J. Webster, K. Nevin, J. Butler, and D.R. Lovley. 2005. DNA microarray analysis of nitrogen fixation and Fe (III) reduction in *Geobacter sulfurreducen*s. Appl. Environ. Microbiol. 71: 2530–2538.

Meyer, F., D. Paarmann, M. D'Souza, et al. 2008. The metagenomics RAST server - a public resource for the automatic phylogenetic and functional analysis of metagenomes. BMC Bioinformatics. 19: 386.

Nevin, K.P., D.E. Holmes, T.L. Woodard, S.F. Covalla, and D.R. Lovley. 2007. Reclassification of *Trichlorobacter thiogenes* as *Geobacter thiogenes* comb. nov. Int. J. Syst. Evol. Microbiol. 57: 463–466.

R-Development-Core-Team. 2008. R: A language and environment for statistical computing. R Foundation for Statistical Computing, Vienna, Austria.

Robinson, M.D., D.J. McCarthy, and G.K. Smyth. 2010. edgeR: a Bioconductor package for differential expression analysis of digital gene expression data. Bioinformatics. 26: 139–140.

Sanford, R.A., J.R. Cole, and J.M. Tiedje. 2002. Characterization and Description of *Anaeromyxobacter dehalogenans* gen. nov., sp. nov., an Aryl-Halorespiring Facultative Anaerobic Myxobacterium. Appl. Environ. Microbiol. 68: 893–900.

Sanford, R.A., D.D. Wagner, Q. Wu, et al. 2012. Unexpected nondenitrifier nitrous oxide reductase gene diversity and abundance in soils. Proc. Natl. Acad. Sci. USA. 109: 19709–19714.

Schmieder, R., and R. Edwards. 2011. Quality control and preprocessing of metagenomic datasets. Bioinformatics. 27: 863–864.

Sung, Y., K.E. Fletcher, K.M. Ritalahti, R.P. Apkarian, N. Ramos-Hernandez, R.A. Sanford, N.M. Mesbah, and F.E. Löffler. 2006. *Geobacter lovleyi* sp. nov. strain SZ, a novel metal-reducing and tetrachloroethene-dechlorinating bacterium. Appl. Environ. Microbiol. 72: 2775–2782.

Takada-Hoshino, Y., and N. Matsumoto. 2004. An improved DNA extraction method using skim milk from soils that strongly adsorb DNA. Microb. Environ. 19: 13–19.

Thomas, S.H. 2009. Ecophysiology and diversity of *Anaeromyxobacter* spp. and implications for uranium bioremediation. Ph.D. thesis. Georgia Institute of Technology, GA, USA.

Urich, T., A. Lanzén, J. Qi, D.H. Huson, C. Schleper, and S.C. Schuster. 2008. Simultaneous assessment of soil microbial community structure and function through analysis of the meta-transcriptome. PloS One. 3: e2527.

Wilke, A., T. Harrison, J. Wilkening, D. Field, E.M. Glass, N. Kyrpides, K. Mavrommatis, and F. Meyer. 2012. The M5nr: a novel non-redundant database containing protein sequences and annotations from multiple sources and associated

tools. BMC Bioinformatics. 13: 141.

Supplementary figure legends

Fig. S1

Seasonal changes of soil redox potential (Eh) and water management. Eh at 5 cm depth was measured using 5 replicate platinum-tipped electrodes as described previously (Itoh *et al*., 2013). Data in 2009 was derived from our previous study (Itoh *et al*., 2013). Asterisks indicate soil samples used for metatranscriptomics in this study.

Fig. S2

Microbial diversity of RNT genes and rRNA gene detected in metagenomics of the present study. Taxonomic distribution of *nar*, *nir*, *nor*, *nos*, *nrf*, and *nif*, and rRNA gene at phylum- and proteobacterial class-level (A), and deltaproteobacterial genus-level (B). Sample IDs indicate data derived from paddy soils in shallow (S1, S3) and deep (S2, S4) layers under waterlogged (S1, S2) and drained (S3, S4) conditions.

Fig. S3

Nif gene clusters conserved in genome of *Anaeromyxobacter* spp. and diazotrophs.

Fig. S1

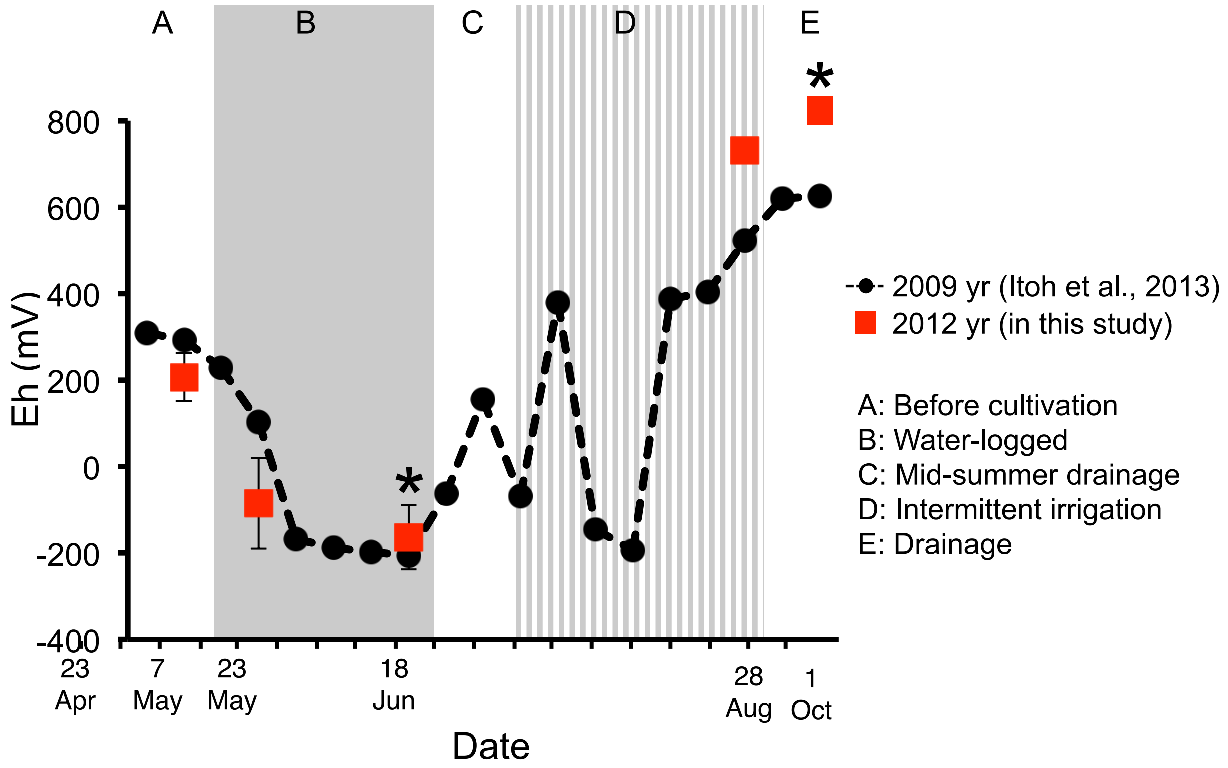

A
B
C
D
E
800
600
400
200
0
-200
-400
Eh (mV)
23 Apr
7 May
23 May
18 Jun
28 Aug
1 Oct
Date
*
*
2009 yr (Itoh et al., 2013)
2012 yr (in this study)
A: Before cultivation
B: Water-logged
C: Mid-summer drainage
D: Intermittent irrigation
E: Drainage

Fig. S2

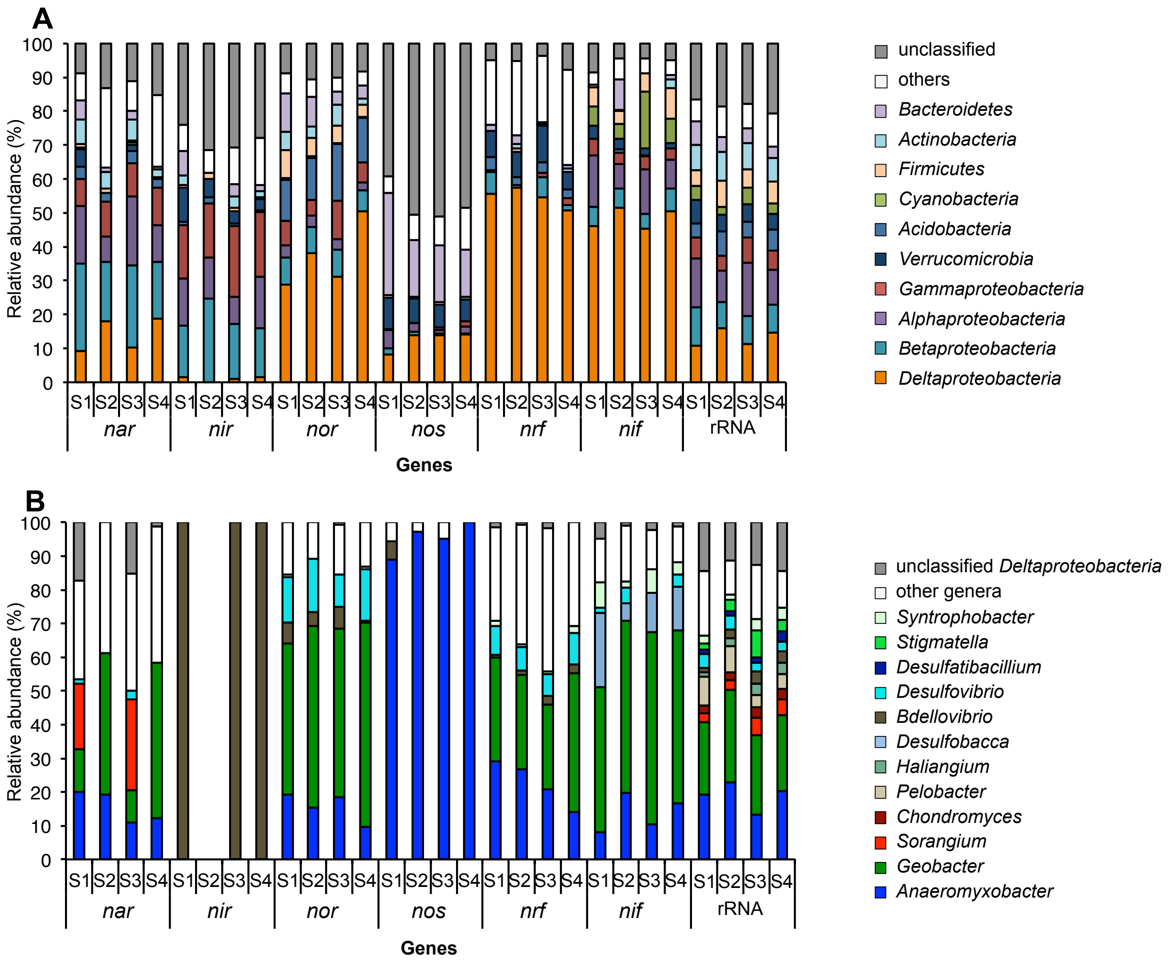

A
Relative abundance (%)
0
10
20
30
40
50
60
70
80
90
100
S1 S2 S3 S4 S1 S2 S3 S4 S1 S2 S3 S4 S1 S2 S3 S4 S1 S2 S3 S4 S1 S2 S3 S4 S1 S2 S3 S4
nar
nir
nor
nos
nrf
nif
rRNA
Genes
unclassified
others
Bacteroidetes
Actinobacteria
Firmicutes
Cyanobacteria
Acidobacteria
Verrucomicrobia
Gammaproteobacteria
Alphaproteobacteria
Betaproteobacteria
Deltaproteobacteria
B
Relative abundance (%)
0
10
20
30
40
50
60
70
80
90
100
S1 S2 S3 S4 S1 S2 S3 S4 S1 S2 S3 S4 S1 S2 S3 S4 S1 S2 S3 S4 S1 S2 S3 S4 S1 S2 S3 S4
nar
nir
nor
nos
nrf
nif
rRNA
Genes
unclassified Deltaproteobacteria
other genera
Syntrophobacter
Stigmatella
Desulfatibacillium
Desulfovibrio
Bdellovibrio
Desulfobacca
Haliangium
Pelobacter
Chondromyces
Sorangium
Geobacter
Anaeromyxobacter

Fig. S3

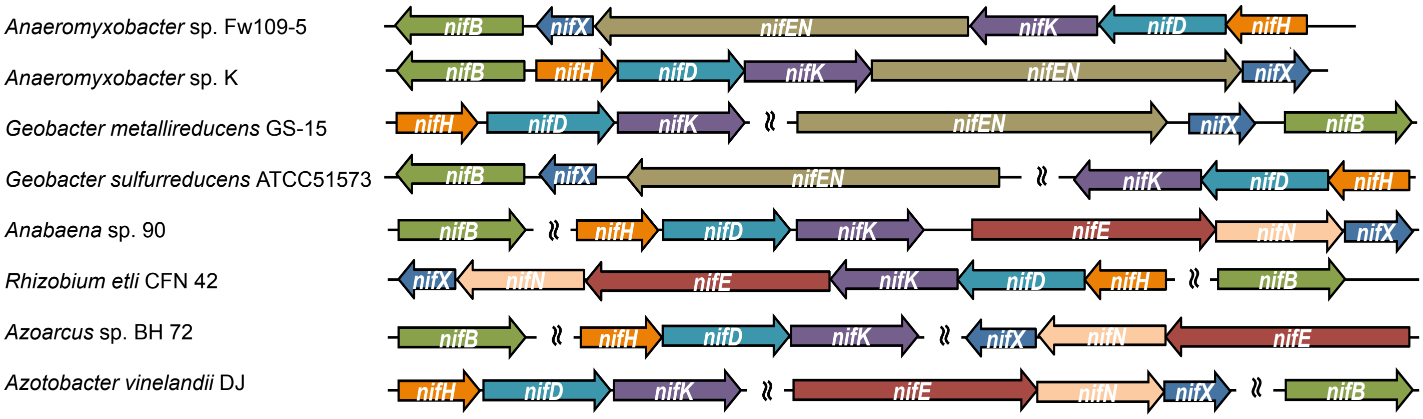

Anaeromyxobacter sp. Fw109-5
nifB nifX nifEN nifK nifD nifH
Anaeromyxobacter sp. K
nifB nifH nifD nifK nifEN nifX
Geobacter metallireducens GS-15
nifH nifD nifK nifEN nifX nifB
Geobacter sulfurreducens ATCC51573
nifB nifX nifEN nifK nifD nifH
Anabaena sp. 90
nifB nifH nifD nifK nifE nifN nifX
Rhizobium etli CFN 42
nifX nifN nifE nifK nifD nifH nifB
Azoarcus sp. BH 72
nifB nifH nifD nifK nifX nifN nifE
Azotobacter vinelandii DJ
nifH nifD nifK nifE nifN nifX nifB

Table S1 RNT activities of *Geobacter* and *Anaeromyxobacter* reported in previous studies.

	References reporitng RNT activities			
Organisms	NOR (NO -> N_2O)	NOS (N_2O -> N_2)	DNRA (NO_3^- -> NH_4^+)	Nitrogen fixation (N_2 -> NH_4^+)
Geobacter	ND[a]	–[b]	Sung et al., 2006; Nevin et al., 2007	Bazylinski et al., 2000; Methé et al., 2005
Anaeromyxobacter	ND[a]	Sanford et al., 2012	Sanford et al., 2002	ND[a,c]

[a] ND, no data.

[b] None of *Geobacter* spp. harbor *nos* in their genome.

[c] *Anaeromyxobacter* sp. FW109-5 and K have similar *nif* cluster to that of *Geobacter metallireducens* GS-15 *and Geobacter sulfurreducens* ATCC51573 having nitrogen fixation activity (Bazylinski et al., 2000, Methé et al., 2005), shown in Fig. S3.

Tabel S2 GC content of *norB, nosZ*, *nrfA*, *nifH*, 16S rRNA gene, and genome of *Anaeromyxobacter* spp. and other genera.

Phylum/Proteobacterial class	Organisms	GC content %						Accession No.
		norB	nosZ	*nrfA*	*nifH*	16S rRNA	genome	
Deltaproteobacteria	*Anaeromyxobacter dehalogenans* 2CP-1	71.2	67.4	70.1	-	58.6	74.8	CP001359
Deltaproteobacteria	*Anaeromyxobacter dehalogenans* 2CP-C	71.2	67.4	69.6	-	58.6	74.9	NC007760
Deltaproteobacteria	*Anaeromyxobacter* sp.Fw109-5	70.6	67.2	68.6	67.4	58.6	73.5	CP000769
Deltaproteobacteria	*Anaeromyxobacter* sp.K	71.1	67.2	70.1	67.8	58.5	74.4	CP001131
Deltaproteobacteria	*Geobacter pickeringii* G13	66.0	-	65.6	61.6	56.4	62.3	CP009788
Deltaproteobacteria	*Geobacter sulfurreducens* KN400	-	-	62.1	60.8	55.9	61.0	CP002031
Deltaproteobacteria	*Geobacter* sp.M21	60.6	-	61.8	61.8	55.0	60.5	CP001661
Deltaproteobacteria	*Geobacter bemidjiensis* Bem	59.6	-	61.0	61.7	55.1	60.3	CP001124
Deltaproteobacteria	*Geobacter metallireducens* GS-15	65.1	-	61.9	58.1	56.4	59.6	CP000148
Deltaproteobacteria	*Geobacter lovleyi* SZ	-	-	49.8	54.4	54.2	54.8	CP001089
Deltaproteobacteria	*Geobacter daltonii* FRC-32	55.5	-	54.5	59.5	54.7	53.5	CP001390
Deltaproteobacteria	*Desulfovibrio vulgaris* Miyazaki F	-	-	61.3	62.2	57.4	67.1	CP001197
Deltaproteobacteria	*Desulfobacterium autotrophicum* HRM2	-	-	-	53.5	52.2	48.8	CP001087
Deltaproteobacteria	*Desulfobacca acetoxidans* DSM 11109	-	-	-	53.7	55.7	52.9	CP002629
Deltaproteobacteria	*Syntrophobacter fumaroxidans* MPOB	56.6	-	59.1	58.9	57.0	59.9	CP000478
Deltaproteobacteria	*Desulfomonile tiedjei* DSM 6799	50.0	53.7	-	50.8	55.9	50.1	CP003360
Bacteroidetes	*Bacteroides cellulosilyticus* ASM 131834v1	-	-	47.6	-	50.5	42.7	CP012801
Gammaproteobacteria	*Shewanella oneidensis* MR-1	-	-	44.9	-	54.8	45.9	NC004347
Gammaproteobacteria	*Escherichia coli* K-12 MG 1655	-	-	52.3	-	54.4	50.8	NC000913
Gammaproteobacteria	*Thioalkalivibrio nitratireducens* DSM 14787	61.0	64.7	62.6	-	56.3	66.5	CP003989
Gammaproteobacteria	*Salmonella enterica, serovar Typhimurium* SL 1344	-	-	54.0	-	54.4	52.2	NC016810
Epsilonproteobacteria	*Sulfurospirillum deleyianum* DSM 6946	-	-	40.7	-	49.2	39.0	CP001816
Epsilonproteobacteria	*Wolinella succinogenes* DSM 1740	-	48.3	49.0	49.0	51.7	48.5	BX571656
Firmicutes	*Clostridium aceticum* ASM 104271v1	-	-	40.3	37.5	54.5	35.3	CP009687
Firmicutes	*Desulfitobacterium dehalogenans* ATCC 51507	45.9	47.4	50.2	53.6	53.9	45.0	CP003348
Cyanobacteria	*Anabaena cylindrica* PCC 7122	-	-	-	42.3	53.7	38.8	CP003659
Cyanobacteria	*Anabaena* sp. 90	-	-	-	44.1	53.3	38.1	CP003284
Cyanobacteria	*Nostoc* sp. PCC 7120	-	-	-	47.4	53.9	41.3	BA000019
Cyanobacteria	*Cyanothece* sp. ATCC 51142	-	-	-	45.1	54.5	38.0	CP000806
Actinobacteria	*Frankia* sp. CcI 3	-	-	-	64.1	60.4	70.1	CP000249
Alphaproteobacteria	*Bradyrhizobium japonicum* USDA 6	59.8	60.9	-	55.0	55.6	63.7	AP012206
Alphaproteobacteria	*Sinorhizobium meliloti* 1021	60.6	60.4	-	58.2	55.4	62.0	NC003047
Alphaproteobacteria	*Rhizobium etli* CFN 42	58.9	-	-	59.8	55.7	61.3	CP000133
Alphaproteobacteria	*Mesorhizobium loti* MAFF 303099	-	-	-	60.5	56.1	62.5	BA000012
Alphaproteobacteria	*Azospirillum thiophilum* ASM 130559v1	62.7	-	-	64.3	57.8	68.2	CP012401
Alphaproteobacteria	*Rhodobacter sphaeroides* ATCC 17025	62.9	65.0	-	63.8	56.0	68.5	CP000661
Betaproteobacteria	*Azoarcus* sp.BH 72	63.8	64.7	-	62.6	55.5	67.9	AM406670
Betaproteobacteria	*Herbaspirillum seropedicae* ASM 104094v1	-	-	-	61.7	53.5	63.4	CP011930
Gammaproteobacteria	*Methylomonas denitrificans* FJG 1	55.1	-	-	49.3	53.3	51.7	CP011476
Gammaproteobacteria	*Azotobacter vinelandii* DJ	-	-	-	59.0	55.8	65.7	CP001157
Gammaproteobacteria	*Klebsiella pneumoniae* 342	61.1	-	-	58.2	54.7	57.3	CP003200

Table S3 Soli samples used in qPCR analyses.

Sample ID	Environment[e]	Collection site	Collection year	Collector	No. of copies/g-sample All bacteria	*Geobacter*	*Anaeromyxobacter*
P1	Paddy	Sapporo, Hokkiado pref.	2011	T. Shinano	$7.11 \pm 0.73 \times 10^{9}$	$2.02 \pm 0.47 \times 10^{8}$	$1.08 \pm 0.18 \times 10^{8}$
P2	Paddy	Kooriyama, Fukushima pref.	2011	H. Itoh	$1.83 \pm 0.22 \times 10^{9}$	$1.72 \pm 0.26 \times 10^{7}$	$1.32 \pm 0.10 \times 10^{7}$
P3[a]	Paddy	Nagaoka, Niigata pref.	2009	H. Itoh	$5.99 \pm 0.98 \times 10^{9}$	$1.36 \pm 0.22 \times 10^{8}$	$8.51 \pm 0.21 \times 10^{7}$
P4[b]	Paddy	Nagaoka, Niigata pref.	2009	H. Itoh	$3.48 \pm 0.91 \times 10^{9}$	$1.15 \pm 0.18 \times 10^{8}$	$2.46 \pm 0.24 \times 10^{7}$
P5	Paddy	Tsukuba, Ibaraki pref.	2011	T. Hasegawa	$3.82 \pm 0.32 \times 10^{9}$	$5.66 \pm 0.61 \times 10^{7}$	$8.30 \pm 1.00 \times 10^{7}$
P6	Paddy	Tanashi, Tokyo pref.	2011	H. Itoh	$1.49 \pm 0.20 \times 10^{9}$	$1.02 \pm 0.14 \times 10^{7}$	$8.57 \pm 0.18 \times 10^{6}$
P7	Paddy	Nagoya, Aichi pref.	2011	S. Asakawa	$4.31 \pm 0.39 \times 10^{9}$	$2.65 \pm 0.15 \times 10^{8}$	$7.03 \pm 0.31 \times 10^{7}$
P8	Paddy	Tsu, Mie pref.	2011	H. Obata	$6.65 \pm 1.03 \times 10^{9}$	$7.30 \pm 0.87 \times 10^{7}$	$7.95 \pm 0.81 \times 10^{7}$
U1	Upland (arable)	Sapporo, Hokkaido pref.	2016	H. Itoh	$3.01 \pm 0.76 \times 10^{9}$	$1.77 \pm 0.10 \times 10^{7}$	$4.21 \pm 1.45 \times 10^{6}$
U2	Upland (grass)	Sapporo, Hokkaido pref.	2016	H. Itoh	$1.61 \pm 0.11 \times 10^{9}$	$5.65 \pm 0.69 \times 10^{6}$	$7.15 \pm 0.29 \times 10^{5}$
U3	Upland (forest)	Sapporo, Hokkaido pref.	2016	H. Itoh	$1.16 \pm 0.06 \times 10^{9}$	$2.92 \pm 0.11 \times 10^{6}$	$5.36 \pm 0.53 \times 10^{5}$
U4[c]	Upland (arable)	Nagaoka, Niigata pref.	2010	H. Itoh	$1.01 \pm 0.19 \times 10^{9}$	$5.34 \pm 0.99 \times 10^{6}$	$4.88 \pm 0.31 \times 10^{6}$
U5[d]	Upland (arable)	Nagaoka, Niigata pref.	2009	H. Itoh	$1.37 \pm 0.29 \times 10^{9}$	$2.47 \pm 0.40 \times 10^{6}$	$3.72 \pm 0.58 \times 10^{6}$
U6	Upland (arable)	Tsukuba, Ibaraki pref.	2011	H. Akiyama	$4.70 \pm 0.30 \times 10^{8}$	$5.81 \pm 0.65 \times 10^{5}$	$5.47 \pm 0.15 \times 10^{5}$
U7	Upland (grass)	Bukyo-ku, Tokyo pref.	2016	Y. Masuda	$3.29 \pm 0.22 \times 10^{9}$	$8.55 \pm 0.42 \times 10^{6}$	$1.07 \pm 0.07 \times 10^{6}$
U8	Upland (arable)	Kanonji, Kagawa pref.	2015	H. Itoh	$1.71 \pm 0.36 \times 10^{9}$	$1.72 \pm 0.11 \times 10^{7}$	$4.06 \pm 2.01 \times 10^{4}$
RS1	River sediment	Joestu, Niigata pref.	2015	H. Itoh	$2.04 \pm 0.20 \times 10^{8}$	$8.20 \pm 0.35 \times 10^{6}$	$2.65 \pm 0.23 \times 10^{6}$
RS2	River sediment	Shunan, Yamaguchi pref.	2015	H. Itoh	$6.85 \pm 0.46 \times 10^{8}$	$1.86 \pm 0.14 \times 10^{7}$	$2.44 \pm 0.13 \times 10^{6}$
RS3	River sediment	Onga-gun, Fukuoka pref.	2015	H. Itoh	$6.23 \pm 0.26 \times 10^{8}$	$2.23 \pm 0.10 \times 10^{7}$	$3.21 \pm 0.19 \times 10^{6}$
RS4	River sediment	Kanzaki, Saga pref.	2015	H. Itoh	$6.56 \pm 0.34 \times 10^{8}$	$1.85 \pm 0.16 \times 10^{7}$	$3.04 \pm 0.21 \times 10^{6}$
RS5	River sediment	Kusu-gun, Oita pref.	2015	H. Itoh	$4.52 \pm 0.56 \times 10^{8}$	$1.27 \pm 0.16 \times 10^{7}$	$2.36 \pm 0.37 \times 10^{6}$
RS6	River sediment	Tamana, Kumamoto pref.	2015	H. Itoh	$1.06 \pm 0.05 \times 10^{9}$	$2.45 \pm 0.15 \times 10^{7}$	$4.05 \pm 0.29 \times 10^{6}$

[a] Collected from the same paddy field used for metatranscriptomics and metagenomics in this study.

[b] Two years after transformed from upland arabele field.

[c] One year after transformed from paddy field, P4.

[d] Two years after transformed from paddy field.

[e] All paddy soils were collected from waterlogged fields.

Table S4 Summary of metatranscriptomics in all soil samples.

Soil ID	Replicate	No. of sequences									MG-RAST ID
		total (length[a] (bp))	rRNA	Protein coding genes	*nar*	*nir*	*nor*	*nos*	*nif*	*nrf*	
S1	1	7,240,814 (175±35)	6,413,363	20,353	0	0	2	0	1	0	4675403.3
	2	6,017,177 (173±37)	5,322,206	17,066	6	1	1	2	2	3	4675404.3
	3	6,968,767 (176±38)	6,171,594	18,163	12	2	1	0	0	3	4675402.3
S2	1	5,667,389 (182±37)	4,897,849	69,750	53	14	22	18	18	24	4678309.3
	2	5,170,274 (186±37)	4,523,846	73,085	66	4	14	14	7	20	4678318.3
	3	6,301,014 (187±36)	5,577,772	85,997	84	8	25	10	13	26	4678319.3
S3	1	5,917,478 (196±35)	5,391,967	22,823	8	0	2	2	2	0	4675332.3
	2	7,284,668 (200±34)	6,595,453	37,417	23	5	5	0	3	4	4675331.3
	3	5,699,284 (191±35)	4,818,110	153,931	107	20	58	28	14	20	4675333.3
S4	1	7,264,805 (187±33)	5,970,105	18,628	17	5	2	13	0	3	4669534.3
	2	5,000,890 (191±34)	3,893,485	13,987	13	2	0	4	1	4	4669537.3
	3	6,969,617 (177±33)	4,304,930	19,777	15	3	6	3	2	0	4669538.3

[a] ave.±std.

Table S5 Summary of metagenomics in all soil samples.

Soil ID	Replicate	No. of sequences									MG-RAST ID
		total (length[a] (bp))	rRNA	Protein coding genes	*nar*	*nir*	*nor*	*nos*	*nif*	*nrf*	
S1	1	1,956,714 (216±48)	3,492	594,728	435	70	125	52	81	59	4670910.3
	2	1,752,612 (240±49)	2,384	672,120	582	78	208	75	96	92	4670906.3
	3	1,442,786 (232±48)	2,242	493,372	427	55	140	66	67	55	4670911.3
S2	1	1,220,590 (255±47)	1,313	514,192	321	45	178	74	136	77	4670909.3
	2	2,016,318 (249±49)	2,048	829,603	457	14	293	123	234	148	4670907.3
	3	371,215 (249±49)	489	142,550	90	14	50	22	44	28	4670904.3
S3	1	1,543,881 (239±49)	1,755	487,420	442	65	108	63	49	39	4670905.3
	2	1,531,818 (247±46)	1,998	660,247	374	81	170	102	48	70	4670900.3
	3	2,029,359 (237±47)	2,092	809,440	649	116	217	121	88	74	4670908.3
S4	1	1,455,107 (248±47)	1,818	590,835	353	92	193	93	110	100	4670902.3
	2	1,265,633 (241±47)	1,315	503,799	288	54	181	86	63	90	4670901.3
	3	1,142,066 (244±48)	1,296	435,977	264	40	141	78	68	64	4670903.3

[a] ave.±std.
